# Regeneration of Granulated Spent Activated Carbon with 1,2,4-Trichlorobenzene Using Thermally Activated Persulfate

**DOI:** 10.1021/acs.iecr.2c00440

**Published:** 2022-06-28

**Authors:** Andrés Sánchez-Yepes, Aurora Santos, Juana M. Rosas, José Rodríguez-Mirasol, Tomás Cordero, David Lorenzo

**Affiliations:** † Departamento de Ingeniería Química y de Materiales, Universidad Complutense de Madrid, Madrid 28040, Spain; ‡ Departamento de Ingenierí́a Química, Universidad de Málaga, Andalucia Tech, Campus de Teatinos s/n, 29010 Málaga, Spain

## Abstract

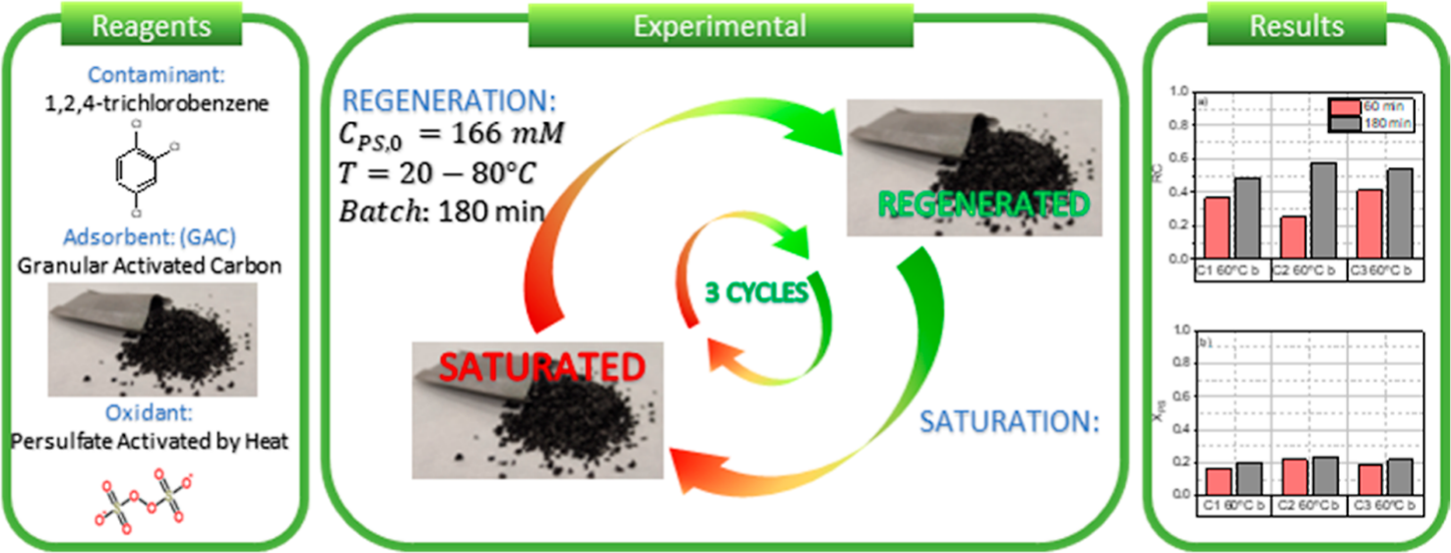

Chlorinated organic compounds (COCs) are persistent organic pollutants often found in groundwater near industrial sites or in industrial wastewaters. Adsorption into activated carbon is a common strategy to remediate these waters, but spent activated carbon results in a toxic residue to manage. To avoid the transport of the chlorinated compounds out of the site, the in-situ regeneration of the spent activated carbon can be considered for reuse to implement a circular economy. In this work, the regeneration of a commercial granular activated carbon (GAC) has been carried out using thermally activated sodium persulfate (TAP). GAC was previously saturated in 1,2,4-trichlorobenzene (124-TCB) as the model compound. The initial adsorption value was 350 mg_124-TCB_·g_GAC_^–1^. First, the nonproductive consumption of sodium persulfate was studied at different temperatures using nonsaturated GAC. Then, the regeneration of the saturated GAC (5 g) was studied by an aqueous solution (166 mM) of TAP (1 L) at a temperature range from 20 to 80 °C. The possible recovery of the adsorption capacity was studied after 3 h of treatment in three successive adsorption–regeneration cycles at the selected temperature (60 °C). The physicochemical changes of the GAC were also investigated before and after the regeneration treatments. The results evidence the significant deposition of sulfate on the GAC after each treatment of regeneration, which avoids the recovery of the initial adsorption capacity. Therefore, each regeneration cycle was necessarily followed by a washing step at 60 °C to remove this sulfate. After that, the regeneration treatment achieved a stable and high recovery of the initial adsorption capacity of about 48.2%.

## 1 Introduction

The presence of chlorinated organic compounds (COCs) in industrial wastewaters and groundwaters is a serious environmental problem. According to the EU Water Framework Directive (Directive 2000/60/EC), several COCs have been added to the list of substances to be monitored in recent decades, limiting their production and use. Examples are 1,1,1-trichloroethane, 1,1,2-trichloroethane, 1,2,4-trichlorobenzene, or 1,4-dichlorobenzene, among others. However, due to their widespread and common use as wood preservatives, pesticides, solvents, hydraulic fluids, or dielectric oil, these COCs still pose an elevated risk to the environment^1−7^ due to their persistent character, so the design of abatement techniques must be accomplished.

One of the most common strategies for treating COCs in polluted groundwater is the groundwater pump and treatment using adsorption into granular activated carbon (GAC) as a hydraulic barrier for this contamination.^8,9^ Activated carbon is also used to treat runoff water collected from contaminated sites. Specifically, GAC is an adsorbent with highly developed porous structures. Its high initial BET surface area close to its amphoteric and hydrophobic properties renders GAC as a suitable adsorbent for retaining organic materials from an aqueous medium.^10^ The latter properties make activated carbon a common strategy for treating wastewaters generated in many processes.^10−16^

However, the high content of COCs in spent GAC makes this adsorbent a highly toxic waste that should be also managed appropriately. Moreover, a remarkable amount of spent GAC waste is produced when a large volume of water needs to be treated. Traditional procedures to manage spent GAC consisting^17−21^ of (i) storing the spent adsorbent in temporary chemical waste landfills or underground storage facilities by burying the waste and (ii) burning this waste in ovens, where all the organic matter will be transformed into CO_2_ and water. However, this latter procedure involved a remarkable risk of production of highly toxic chlorinated species (as dioxins) during burning, due to the presence of chlorinated compounds in GAC. On the other hand, the storage of such toxic waste is also against sustainable environmental policies.

Recently, the chemical regeneration of the spent GAC by advanced oxidation processes has been gaining attention for the reuse of spent GAC, minimizing environmental impacts, increasing the circular economy of the process, and reducing the volume of spent adsorbent material waste.^22^ COCs are removed from the aqueous medium, and the adsorbent is regenerated and reused, preferably in the same location.^23,24^

For this purpose, sodium persulfate (PS) activated by temperature (TAP) was studied as an oxidant in this work. Thermal activation of PS generates the sulfate radical, SO_4_^–•^, which has a redox potential of 2.6 V^25−28^ according to eq 1.
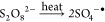
1

In the literature, few works deal with temperature-activated PS (TAP) for the regeneration of spent GAC, used for the removal of pollutants from aqueous streams, neither of them from chlorinated contaminants. In this sense, Jatta et al.^27^ investigated the regeneration of a spent activated carbon saturated in toluene, with 211 mg·g_AC_^–1^. In the regenerative process, they achieved 90% char recovery when applying a 100 mM PS solution at 80 °C. This degree of regeneration was stable during three consecutive regeneration cycles. Similarly, Huling et al.^29^ studied the recovery of a GAC saturated in MTBE after removal of MTBE from an aqueous stream, achieving a saturation of 44.9 mg·kg_GAC_^–1^. The GAC recovery achieved was 40.9% after a single contact cycle with a 40 g·L^–1^ PS solution at 55 °C. Despite the interest in results shown in these cited works, little attention was paid to the changes in the adsorbent through different cycles of regeneration with TAP.

This work has selected 1,2,4-trichlorobenzene (124-TCB) as a model organochlorine pollutant.^2,30,31^ This COC is a semivolatile compound with a moderate water solubility (28 ppm). Moreover, due to its toxicity and persistence, this pollutant poses a risk to human health and environmental safety.^2,3,5,6^ In previous studies, different recalcitrant compounds such as chlorobenzenes in the aqueous phase were degraded by TAP.^25,26^ Specifically, for 124-TCB, the complete mineralization reaction by application of TAP is shown in eq 2:
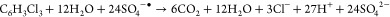
2

The main scope of this work is to analyze the regeneration of a GAC saturated with 124-TCB by TAP in successive adsorption–regeneration cycles. Moreover, attention will be paid to GAC physicochemical changes after the saturation and regeneration cycles. To our knowledge, this is the first time that GAC properties (porosity and surface chemistry) during the intermediate stages of the regenerative process have been evaluated.

## 2 Materials and Methods

### 2.1 Materials

Analytical-grade 1,2,4-trichlorobenzene (124-TCB) was purchased from Sigma-Aldrich. Pure 124-TCB was dissolved in *n*-hexane to plot the calibration curves. Quantification of the contaminants in the reaction samples was carried out using bicyclohexyl as a standard internal compound (ISTD), also purchased from Sigma-Aldrich.

The original (GAC-O) has a BET surface area of 905 m^2^·g^–1^ and a total pore volume of 0.42 cm^2^·g^–1^. Initially, GAC was previously washed with acidic water (GAC-F) as follows: 5 g of GAC was placed in a mesh and left in 1 L of Milli-Q water at pH = 3 for 24 h, with magnetic agitation. The mesh with GAC-F was recovered, rinsed twice with Milli-Q water, and dried 24 h at 50 °C in an oven. After this procedure, the GAC-F surface area and pore volume were reduced to 871 m^2^·g^–1^ and 0.39 cm^2^·g^–1^, respectively. The GAC-F was used as the adsorbent to conduct the adsorption–regeneration experiments.

Sodium PS, used as an oxidant, was supplied by Sigma-Aldrich. Other reagents used to quantify PS, such as potassium iodide (KI) and sodium bicarbonate (NaHCO_3_), were also supplied by Sigma-Aldrich. The concentration of chloride and short-chain organic acids was determined by ion chromatography. The following compounds were used: sodium carbonate, sodium bicarbonate, sulfuric acid, and acetone (all from Sigma-Aldrich).

### 2.2 124-TCB Adsorption

GAC (2 g) was placed in a stainless-steel mesh and immersed in the upper part of a 1 L closed flask containing 800 mL of water (Milli-Q purity), following which 3 g of pure 124-TCB was added as a liquid organic phase. A scheme of the experimental setup used is given in Figure S1 of the Supporting Information. The multiphase medium (organic–aqueous–GAC) was stirred to ensure that no external diffusional resistances were present. The amount of 124-TCB added was sufficient to saturate the GAC, with some of the added TCB remaining as an undissolved organic phase. In this way, the solubilized 124-TCB in water was kept at 28 mg·L^–1^ (solubility of pure 124-TCB in water at 25 °C) during the whole adsorption time, thanks to the excess of 124-TCB organic phase present. Equilibrium between 124-TCB in the aqueous phase (28 mg·L^–1^) and 124-TCB adsorbed on the GAC was reached before 72 h. After this time, the mesh with GAC was withdrawn and rinsed with water twice, dried at 50 °C for 24 h, and used in the regeneration experiments.

The excess of 124-TCB remaining in an organic phase and the 124-TCB solubilized in the aqueous phase was extracted by adding 200 mL of hexane to this medium containing the organic and aqueous phases. The mixture was stirred for 60 min to ensure the complete extraction of 124-TCB in hexane. GC–MS quantified the total concentration of 124-TCB extracted in hexane. The mass of 124-TCB adsorbed on GAC was calculated as the difference between the initial 124-TCB added as an organic phase and the final 124-TCB extracted in hexane. This procedure was accomplished in triplicate, and differences lower than 10% were obtained.

### 2.3 Experimental Procedure

Porosity and surface chemistry of original and pretreated GAC were characterized by adsorption–desorption of N_2_ at −196 °C, X-ray photoelectron spectroscopy (XPS), and temperature-programed desorption (TPD), as described in the next sections.

Two sets of experiments were carried out: using the pretreated activated carbon without contaminant (GAC-F) and the saturated one in 124-TCB (GAC-S).

#### 2.3.1 Reactivity of TAP with GAC-F

In the first set of experiments, the reaction between PS and GAC-F was studied batchwise at different temperatures (20–80 °C) and GAC loadings (0–10 g·L^–1^). A 50 mL closed glass flask containing 50 mL of Milli-Q water was immersed in a thermostated glycerin bath. Constant temperature and good agitation were achieved using a mixing plate (IKA C-MAG HS 7). Once the temperature was reached, 1.97 g of PS was added to obtain a concentration of 166 mM in the aqueous phase, and a mass (0, 0.25, or 0.5 g) of pretreated GAC was confined in a stainless-steel mesh basket and introduced in the aqueous PS solution (zero reaction time). The GAC loading corresponds to 0, 5, and 10 g_GAC_·L^–1^ in the batch reactor, respectively. Experimental conditions are summarized in Table 1. A volume of 0.10 mL of the aqueous phase sample was taken at several reaction times to quantify the PS concentration. At the final reaction time (6 h), the mesh with the GAC was recovered and treated in two different ways

a. Used directly in the following cycle (runs B8, B9, B11, and B12) and carried out under the corresponding reaction conditions.
b. Washed in a 50 mL agitated flask with 50 mL of Milli-Q water at 60 °C for 2 h and used in the following cycle (runs B8 and B9), under the corresponding reaction conditions.

**Table 1:** Experimental Conditions for Runs Carried out Using GAC-F without COCs Adsorbed^a^

run	*T* (°C)	*C*_GAC-F,0_ (g·L^–1^)	cycle	procedure
B1	20	0	1	
B2	20	5	1	
B3	20	10	1	
B4	40	0	1	
B5	40	5	1	
B6	40	10	1	
B7	60	0	1	
B8	60	5	1,^b^ 2, 3^b^	a,^b^ b^b^
B9	60	10	1, 2, 3	a, b
B10	80	0	1	
B11	80	5	1, 2, 3	a, b
B12	80	10	1, 2, 3	a, b

a *C*_PS,0_ = 166 mM.

b The GAC after these cycles was characterized by adsorption–desorption of nitrogen at −196 °C, XPS, and TPD.

The number of cycles and type of procedure for each run are also summarized in Table 1. Samples of GAC obtained in the B8 run after the first and third cycles in procedure b were also characterized after washing at 60 °C with water for 2 h and then were dried at 50 °C in an oven for 24 h.

#### 2.3.2 Regeneration of GAC-S

In the second set of experiments, the GAC-S regeneration by TAP was studied. The pretreated GAC was saturated in 124-TCB (selected as the model COC).

The runs (summarized in Table 2) were conducted batchwise in a 50 mL magnetically stirred reactor. A 50 mL closed glass flask containing 50 mL of Milli-Q water was immersed in a thermostated glycerin bath controlled by a proportional–integral–derivative controller coupled with a heating and mixing plate (IKA C-MAG HS 7) to maintain a constant temperature. Once the temperature was reached, the required amount of PS was added to the aqueous phase. A mass (0.25 g) of GAC (saturated in 124-TCB or not) was confined in a stainless-steel mesh basket and introduced in the aqueous PS solution (zero reaction time). The mass of GAC added corresponds to a loading of 5 g_GAC_·L^–1^ in the batch reactor.

**Table 2:** Experimental Conditions of Runs Carried Out for the Regeneration of GAC-S with TAP, *C*_PS,0_ = 166 mM, and GAC Loading of 5 g·L^–1^^a^

run	*T* (°C)	regeneration cycles	resaturation cycles	procedure
R1	20	1	1	a, b
R2	40	1	1	a, b
R3	60	1,^b^ 2, 3^b^	1, 2, 3	a(1, 2), b(1, 2, 3)^b^
R4	80	1	1	a, b

a *V*_L_ = 50 mL, GAC-S = 0.25 g.

b The GAC was characterized after regeneration, after water rinsing, and, finally, after resaturation.

Several flasks were used in each experiment, a flask being sacrificed each time. Experiments were carried out in duplicate or triplicate. At the corresponding time, the mesh with GAC was withdrawn from the aqueous phase, and the remaining PS and pH in the aqueous phase were determined. The recovered GAC samples were analyzed to determine the recovery of GAC adsorption capacity after oxidation. The analysis of the recovered GAC adsorption capacity after regeneration was evaluated by saturating the oxidized GAC again in a 124-TCB aqueous solution (28 mg·L^–1^) in the following way: (1) Regenerated GAC recovered in the basket mesh (0.25 mg) was placed (with or without previous washing with water at 60 °C) in a 50 mL closed glass flask containing 40 mL of Milli-Q water and 125 mg of 124-TCB (as an organic phase to ensure the saturation of 124-TCB in the aqueous phase at 28 mg·L^–1^). The vial was agitated (300 rpm) for 72 h. After 4 h of settling, the mesh containing the GAC was withdrawn. (2) A volume of 10 mL of hexane was added to the flask, and the liquid phases were agitated for 60 min. After settling for 20 min, GC–MS was used to analyze the concentration of 124-TCB in the hexane phase. (3) The recovery of the adsorption capacity (RC) was calculated using eq 3:
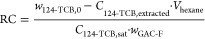
3where *w*_124-TCB,0_ is the mass of 124-TCB added to saturate the oxidized GAC (in mg), *C*_124-TCB,extracted_ is the 124-TCB concentration remaining in *n*-hexane in mg·L^–1^, *C*_124-TCB,sat_ is the concentration of 124-TCB in the saturated GAC-F (mg_124-TCB_ g_GAC-F_^–1^), *w*_GAC-F_ is the mass of GAC-F added, and *V*_*n*-hexane_ is the volume of hexane used in extraction in liters.

The mass of *w*_GAC-F_ must be calculated from the mass of saturated GAC added, *w*_GAC-S_, after the mass of 124-TCB adsorbed is subtracted.
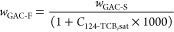
4

Three consecutive regeneration and adsorption cycles were performed for GAC under the reaction conditions used in run 3.

As commented previously, the recovered mesh with GAC after the oxidation treatment was handled in two ways before resaturation in 124-TCB:

a. Without washing with water before resaturation in 124-TCB (run 3).
b. Washing in a 50 mL agitated flask with 50 mL of Milli-Q water at 60 °C for 2 h (run 3).

The GAC was characterized by adsorption–desorption of nitrogen at −196 °C, XPS, and TPD after regeneration (GAC-R) after water rinsing (GAC-R-W) and finally after resaturation (GAC-R-W-S). Schemes of the experimental procedure are provided in the Supporting Information (Figures S1 and S2).

### 2.4 Analytical Methods

Quantification of 124-TCB and identification of other organic byproducts were performed by gas chromatography (Agilent 6890N, Santa Clara, CA, USA) using a mass spectrometry detector. An HP-5MS chromatographic column (30 m × 0.25 mm ID × 0.25 μm) was used as the stationary phase and helium at a constant flow rate of 1.7 mL·min^–1^ as the mobile phase. A 1 μL aliquot of the liquid sample was injected (injection port temperature: 250 °C). The chromatographic oven was operated under a programed temperature gradient (initial temperature = 80 °C, increasing the temperature at a rate of 18 °C·min^–1^ up to 180 °C, and then keeping it constant for 15 min). Bicyclohexyl was used as a standard internal compound (ISTD), 8 mg·kg_hexane_^–1^.

The concentration of PS was determined by colorimetric titration using an indicator solution of KI (100 g·L^–1^) and NaHCO_3_ (5 g·L^–1^). Ionic organic byproducts, such as carboxylic acids and chlorides, were measured by ion chromatography (Metrohm 761 Compact IC), with anionic chemical suppression and a conductivity detector. The pH was measured with a basic 20-CRISON pH electrode.

The porous texture of the samples was characterized by N_2_ adsorption–desorption at −196 °C and by CO_2_ adsorption at 0 °C performed using an ASAP 2020 apparatus (Micromeritics). Samples were outgassed at 150 °C for at least 8 h. From the N_2_ isotherm, the apparent surface area (*A*_BET_) was determined by applying the BET equation. The t-method allows obtaining the values of the external surface area (*A*_t_) and the micropore volume (*V*_t_). The mesopore volume (*V*_mes_) was determined as the difference between the adsorbed volume of N_2_ at a relative pressure of 0.99 (*V*_tot_) and the micropore volume *V*_t_. The Dubinin–Radushkevich equation was used to calculate the apparent surface area (*A*_DR_) and narrow micropore volume (*V*_DR_) from CO_2_ adsorption data.

The surface chemistry of the sample was analyzed by XPS (5700C model Physical Electronics) using Mg kα radiation (1253.6 eV). The maximum C 1s peak was set to 284.5 eV and used as a reference for shifting the whole spectrum.

TPD is usually used to characterize the oxygen functional groups present on the carbon surface, which are formed during carbonization/activation processes. TPD analyses were carried out using a customized quartz fixed-bed reactor placed inside an electrical furnace and coupled to both a mass spectrometer (Pfeiffer Omnistar GSD-301) and to a nondispersive infrared (NDIR) gas analyzer (Siemens ULTRAMAT 22) to quantify CO and CO_2_ evolution (calibration error <1%). In these experiments, ca. 100 mg of GAC was heated from room temperature to 930 °C, at a heating rate of 10 °C·min^–1^ under nitrogen (purity 99.999%, Air Liquide) flow (200 cm^3^·STP·min^–1^).

## 3 Results and Discussion

### 3.1 GAC Characterization

The textural parameters of original carbon (GAC-O) and that washed with acid water (GAC-F) are summarized in Table S1. The N_2_ adsorption–desorption isotherms obtained at −196 °C of both samples are given in Figure S3 of the Supporting Information The atomic surface concentration was determined by XPS analyses, and the amount of CO and CO_2_ evolved from TPD experiments for these samples are also collected in Table S1.

As shown in Figure S3, GAC is a microporous material presenting an isotherm of type I.^32^ GAC presented an initial apparent surface area of 905 m^2^·g^–1^, which was reduced by 4% after its washing (GAC-F). An equivalent reduction of the pore volume (7%) was observed after washing it with acid water. With regard to the surface chemistry, GAC presented an atomic surface concentration of oxygen of around 10% and traces of N and S. The content of S was significantly reduced by the washing process. Meanwhile, the amount of CO and CO_2_ evolved from TPD are not very significant, evidencing the low presence of carbon–oxygen surface groups. As can be seen, these amounts slightly increased after washing, mainly associated with the higher decomposition of carbonyl and quinone groups.

### 3.2 PS Consumption by GAC-F

The effect of the reaction between GAC without the contaminant adsorbed (GAC-F) and PS was deeply investigated at different reaction temperatures, studying the PS consumption and the changes in the carbon properties.

The consumption of PS promoted by GAC-F was tested at different reaction temperatures and using different concentrations of GAC and PS. The experimental conditions are summarized in Table 1. The PS concentration was measured, and the conversion of PS was calculated using eq 5:
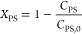
5where *C*_PS,0_ and *C*_PS_ are the initial concentration of PS and the concentration for a specific reaction time, respectively.

**Figure 1:**
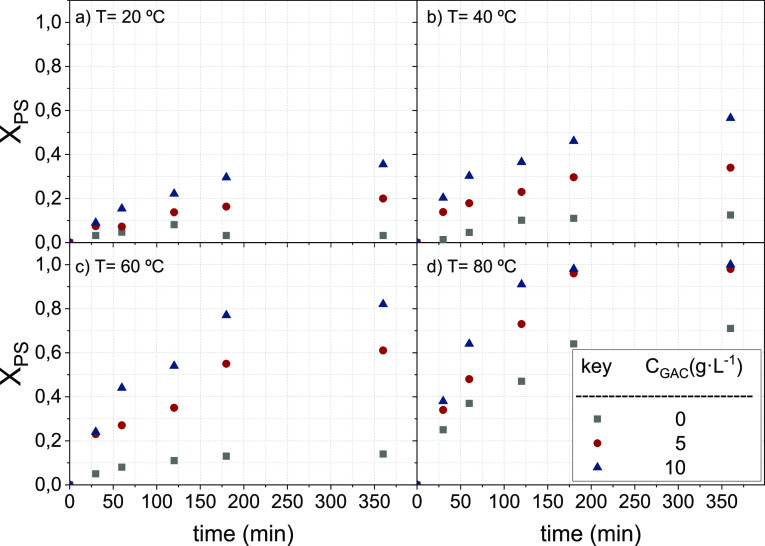
PS conversion profiles with reaction times in the first cycle (runs in Table 1) at different GAC-F concentrations and *C*_PS,0_ = 166 mM at (a) 20, (b) 40, (c) 60, and (d) 80 °C, respectively.

The profiles of PS conversion using GAC-F in the first cycle of runs carried out under the experimental conditions in Table 1 are shown in Figure 1. The higher the temperature, the higher the PS consumption, as expected in TAP. This trend was noticed with or without GAC in the medium. In GAC’s absence, the PS consumption was negligible in the interval time studied in the temperature range of 20–60 °C (conversion lower than 0.15). On the contrary, consumption of PS rises at 0.71 at 360 min at 80 °C. These values are in agreement with those reported elsewhere.^26^

The presence of GAC results in a remarkable increase in PS consumption, suggesting that PS decomposed at the GAC surface. The higher the GAC loading, the higher the PS conversion at a specific time, as shown in Figure 1. These differences were more significant at temperatures lower than 80 °C, suggesting that the activation energy of the heterogeneous reaction was lower than the activation energy of the homogeneous thermal reaction or the adsorption of PS into the carbon surface, especially at the lowest temperature.

In addition, the effect of the mesh in the reaction medium was elucidated. Experiment B8 was carried out using the GAC dispersed in the aqueous phase. The differences between the PS consumption with and without mesh were negligible (<2%).

The stability of GAC-F after oxidation with TAP was analyzed. Reactivity and structural changes of GAC caused by its reaction with PS were studied after different reaction cycles, using 5 and 10 g·L^–1^ of GAC, at 60 and 80 °C (runs B8, B9, B11, and B12 in Table 1). At the end of each cycle (180 min), the GAC was separated, dried, and put in contact with a new solution of PS (*C*_PS,0_ = 166 mM), with or without previous washing with water, as explained in the Experimental Procedure section. The final pH reached after each cycle was 1.5 due to PS decomposition, resulting in acidic pH.

The time profiles of PS conversion obtained at each cycle, without washing the GAC recovered after TAP oxidation (procedure a), are shown in Figure S4 of the Supporting Information. As shown in Figure S4, the PS conversion decreased with the cycle regardless of the temperature and GAC concentration used. At both concentrations tested, the PS consumption decreased dramatically between cycles 2 and 3. The drop in PS conversion in successive cycles could be due to the salt deposition on the surface of GAC.^33^ The generated inorganic salts (sulfates) in the aqueous medium can be deposited on the GAC surface, hindering the PS reaction on the GAC surface. Results obtained in run 3 with and without GAC washing between cycles (at 60 °C, procedure b in the Experimental Procedure section) are compared to confirm this hypothesis. The PS conversion with time is shown in Figures S4a and S5a.

**Figure 2:**
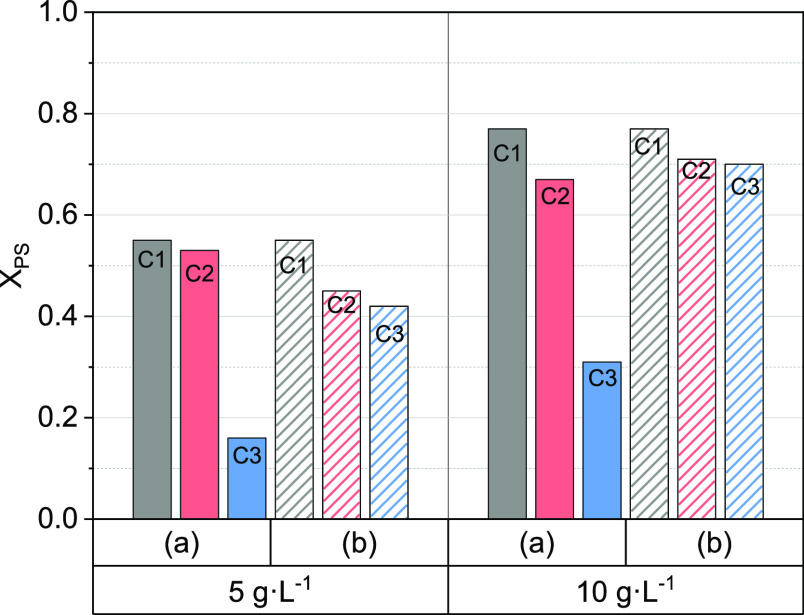
PS conversion at 180 min runs B8 and B9 in Table 1, with (a) and without (b) washing with water at 60 °C between oxidation cycles. PS conversions profiles with time are shown in Figures S4a,b and S5a,b.

In Figure 2, PS conversion measured at each cycle in runs B8 and B9 (Table 1), with and without previous washing with water at 60 °C, are compared. As shown in Figure 2, if GAC is washed between oxidation cycles, the conversion of PS is stable over cycles, confirming that the deposition of inorganic salts on the GAC surface decreases the GAC reactivity with TAP.

Changes in the GAC-F porosity and BET area were measured after several cycles in run B8 (Table 1) carried out with procedure a (without washing) or b (washing with water for 2 h, at 60 °C, between the different cycles). The BET surface area and the pore volume were measured after the first cycle (GAC-B8-C1a) and after the third cycle (GAC-B8-C3b), and the results are given in Table 3. The corresponding nitrogen adsorption–desorption isotherms at −196 °C are summarized in Figure S6.

**Table 3:** Physicochemical Changes in GAC-F after Reaction with TAP; Run B8 in Table 1

analytical technique	property	GAC-F	GAC-B8-C1a	GAC-B8-C3b
N_2_ adsorption	*A*_BET_ (m^2^·g^–1^)	871	737	173
	*V*_P_ (cm^3^·g^–1^)	0.39	0.36	0.09
CO_2_ adsorption	*A*_DR_ (m^2^·g^–1^)	459	409	115
	*V*_DR_ (cm^3^·g^–1^)	0.184	0.164	0.05
XPS: atomic surface concentration	C (%)	90.59	78.79	69.16
	N (%)	0.51	0.88	0.63
	O (%)	8.75	18.85	24.75
	S (%)	0.15	1.47	0.36
TPD	CO (μmol·g^–1^)	349	3058	3068
	CO_2_ (μmol·g^–1^)	41	1405	3557
	H_2_O (μmol·g^–1^)	73	404	940

The BET surface area of the GAC-F before oxidation was 871 m^2^·g^–1^ (Table S1). In GAC-B8-C1a (after the first oxidation cycle), the apparent surface area decreased by 15%. In addition, after the third oxidation cycle GAC-B8-C3b, the surface area drops to a value of 173 m^2^·g^–1^, associated with 76% of porosity loss or blockage. The reaction between GAC-F and PS can explain this decrease in the specific surface area. PS oxidizes the surface, modifying the GAC textural parameters, reducing the available pores, and diminishing the accessible surface area. These data are consistent with the results obtained by XPS and TPD, where generation of many oxygen surface groups was observed.

The reaction of TAP with GAC-F also changed the chemical composition of the GAC-F surface. As shown in Table 3, the atomic surface concentration of carbon was reduced during the cycles, favoring the formation of more oxidized surface groups, as confirmed by the high increase of the oxygen surface concentration after the different cycles determined by XPS and TPD. This oxygen increase is more significant in XPS analyses, suggesting that PS treatment strongly affects the most external surface. Specifically, the evolved CO, CO_2_, and H_2_O amounts from TPD analyses significantly increased with the reaction cycles. The range of evolution of these molecules is associated with the decomposition of carboxylic acids, lactones, anhydrides, and phenols/ether groups, not observed in the fresh GAC (GAC-F). In fact, after the third cycle, the amount of evolved CO_2_ considerably increased, mainly due to further formation of carboxylic acids, lactone, and anhydride groups. The increase of acid groups after GAC-F’s contact with PS inferred the following mechanism of carbon oxidation, proposed in eqs 6 and 7:
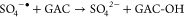
6
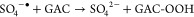
7The sulfate radical obtained by the thermal activation of PS defined in eqs 6 and 7 attacks the GAC surface, generating the above-mentioned functional groups.

Finally, the S surface concentration determined by XPS analyses increased after reaction with TAP, as can be seen in GAC-B8-C1a, shown in Table 3, probably due to sulfate deposition. This deposition was removed after the washing steps, as evidenced in the results for GAC-B8-C3b, shown in Table 3. The initial S % content in GAC-F is 0.15, which increased to 1.47 after one cycle with TAP and decreased to 0.36 after washing with water at 60 °C, suggesting the effectiveness of hot washing in the sulfate removal. The latter was consistent with the experimental results obtained by IC where sulfates were detected.

### 3.3 Regeneration of GAC Saturated in 124-TCB

The saturation of GAC-F in 124-TCB (28 mg·L^–1^ in the aqueous phase), explained in the Experimental Procedure, resulted in a GAC with 350 mg_124-TCB_·g_GAC-F_^–1^ (*C*_124-TCB,sat_ in eq 4).

The procedure to study the recovery of GAC adsorption capacity (RC) by oxidation of GAC-S with TAP has been described in Section 2.3.2. Runs carried out are summarized in Table 2, and RC is calculated with eq 3.

#### 3.3.1 Adsorption Capacity Recovery without Washing of Regenerated GAC

**Figure 3:**
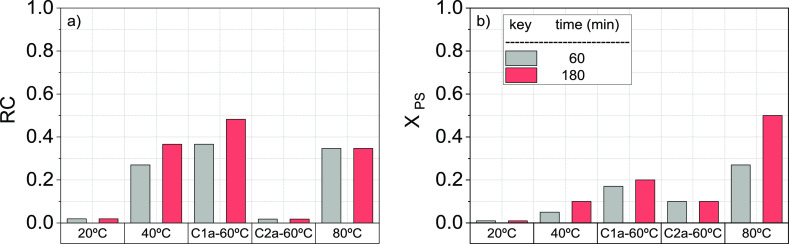
Experimental results of (a) regeneration capacity (RC) calculated with eq 3 of GAC-S and (b) PS conversion (eq 5) at different reaction times, using *C*_PS,0_ = 166 mM and*C*_GAC-S_ = 5g·L^–1^. Runs in Table 2. Without GAC washing after regeneration and before resaturation for one cycle of regeneration.

RC values obtained in the first regeneration cycle of runs R1 to R4 in Table 2, without washing with water (procedure a in Section 2.3.2) between regeneration and resaturation steps, are shown in Figure 3a, for 60 and 180 min. The corresponding consumption of PS in the regeneration step is also shown in Figure 3b.

As shown in Figure 3a, the RC was negligible at 20 °C, agreeing with the negligible PS consumption shown in Figure 3b. On the other hand, partial regeneration of the GAC-S was noticed for temperatures higher than 40 °C. As can be seen in Figure 3, the higher the temperature, the higher the PS consumption. The regeneration obtained at 80 °C (0.35) was lower than that obtained at 60 °C (0.48). Moreover, RC at 80 °C remained constant with the reaction time.

In this sense, the results shown in Figure 3a were obtained without GAC washing, between regeneration and resaturation in 124-TCB. Therefore, the RC values shown in Figure 3a can be affected by the sulfate formation on the GAC surface. This formation is higher at 80 °C due to the higher PS consumption at this temperature.^33^ Moreover, the higher the PS conversion, the lower the final pH of the aqueous solution (2.07, 1.24, and 1.05 for 40, 60, and 80 °C, respectively).

The efficiency of PS consumption for 124-TCB oxidation on the GAC surface was defined as *R*_OX_, this parameter being calculated from eq 8 as the ratio between the moles of PS consumed to the amount of 124-TCB oxidized (assuming that this last value is the same as the millimoles of 124-TCB adsorbed in resaturation of the regenerated GAC):
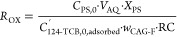
8where *C*_PS,0_ is the initial concentration of PS used (166 mM), *V*_AQ_ is the aqueous volume of the aqueous phase in the reaction medium (0.05 L), *C*_124-TCB,0,adsorbed_^′^ is the millimoles of 124-TCB adsorbed in the initial saturated GAC-F (2.8 mmol·g^–1^), *w*_GAC-F_ the amount of GAC-F added, calculated from eq 4, and *X*_PS_ is the PS conversion.

**Figure 4:**
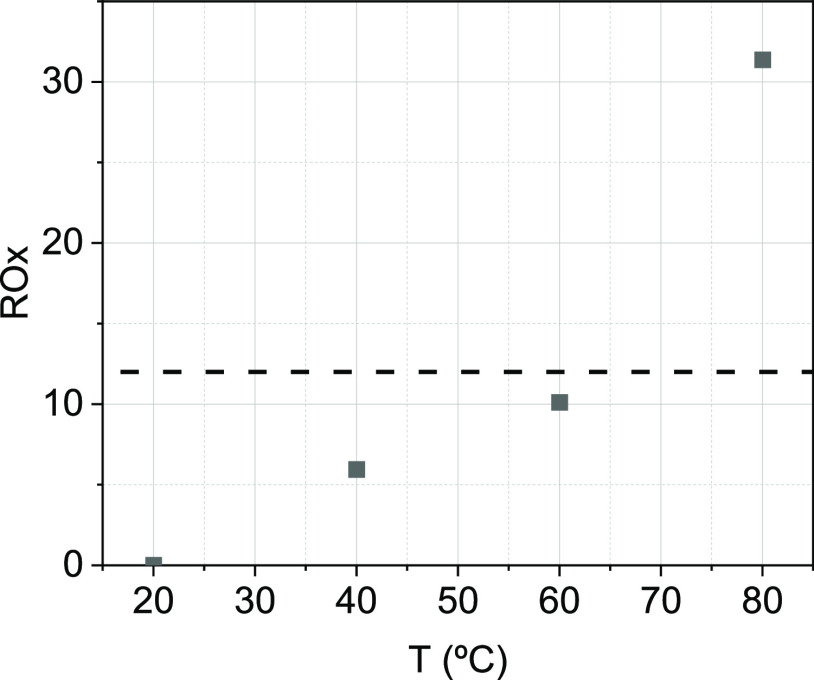
Oxidation yield, *R*_OX_ in *l*_PS,reacted_·mmol_124-TCB,regenerated_^–1^, for the experiments R1–R4 in Table 2, calculated using eq 8, in the first cycle of regeneration–resaturation without GAC washing between both steps.

The values of *R*_OX_ obtained in experiments R1–R4 (Table 2) are plotted in Figure 4. The higher the *R*_OX_ value, the lower the PS efficiency in 124-TCB oxidation and the higher the unproductive PS consumption. The theoretical value of *R*_OX_ for 124-TCB mineralization is close to 12 according to eq 2. A *R*_OX_ value lower than 12 means that the total mineralization of 124-TCB is not achieved, and a higher value of 12 implies a higher PS consumption. The closest value to 12 of *R*_OX_ was obtained at 60 °C (10.1 mmol_PS,reacted_·mmol_124-TCB,regenerated_^–1^). These conditions can be established as the best temperature to regenerate the GAC-S.

Once selected *T* = 60 °C as the best temperature tested, two cycles of regeneration–resaturation were carried out under the conditions of run R3 in Table 2. The regenerated GAC was not washed with water before resaturation in 124-TCB. The RC and PS conversion in the second regeneration–resaturation cycle are also included in Figure 3. As can be seen, a dramatic decrease in RC was noticed in the second regeneration cycle in R3 (Table 2) when the GAC was used directly in cycles without washing, between regeneration and resaturation steps. The *R*_OX_ for this experiment was 121 mmol_PS,reacted_·mmol_124-TCB,regenerated_^–1^ due to the abatement of 124-TCB was negligible.

#### 3.3.2 Adsorption Capacity Recovery with Washing of Regenerated GAC

**Figure 5:**
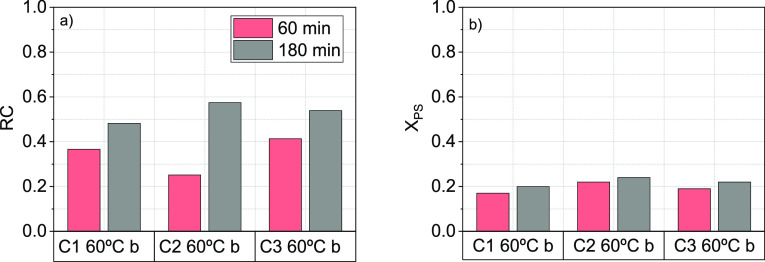
RC (eq 3) and PS conversion (eq 5) of GAC-S after three regeneration/adsorption cycles with washing between GAC regeneration and 124-TCB resaturation. Experimental conditions (run R3, Table 2), with *C*_PS,0_ = 166 mM,*C*_GAC-S_ = 5 g·L^–1^, *T* = 60 °C.

The effect of GAC washing with water between regeneration and resaturation was studied at the selected temperature (60 °C). Three cycles of GAC regeneration and resaturation were carried out under the conditions of run 3 (Table 2), using procedure b (Section 2.3.2). Experimental values for RC (eq 3) and PS conversion (eq 5) at 60 and 180 min are shown in Figure 5, respectively. As shown, RC increases with time. An RC value of about 50% was held in the successive regeneration–adsorption cycles.

The *R*_OX_ value after each cycle was calculated with the data plotted in Figure 5, and the corresponding values obtained were 10.01, 9.09, and 8.88 mmol_PS,reacted_·mmol_124-TCB,regenerated_^–1^ for the first, second, and third cycles, respectively.

Values of *R*_OX_ lower than 12 suggest that the total mineralization of 124-TCB was not achieved. However, GC–MS or GC-FID-ECD identified neither chlorinated nor aromatic compounds in the aqueous phase. In addition, the aqueous phase after washing GAC with hot water was analyzed by two different methods. In the first one, *n*-hexane was used to extract the organic compounds; in the second, water was analyzed by HPLC. Both methods detected neither organic compound.

In addition, the chloride concentration in water after each cycle of regeneration step of the GAC was quantified. The experimental Cl^–^ ((Cl^–^)_exp_) concentration was compared to the theoretical amount of Cl^–^ that should be generated for the total dichlorination of 124-TCB ((Cl^–^)_stq_ calculated with eq 9):
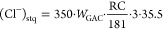
9where 350 mg·g^–1^ is the total amount of 124-TCB adsorbed and 181 and 35.5 are the mass weights of 124-TCB and chloride, respectively.

The experimental chloride concentrations obtained after the three cycles were 17, 20, and 19%, respectively, of the theoretical values expected for total dechlorination of the oxidized 124-TCB. These low values can be explained by assuming the formation of more oxidized chlorinated species that were not detected^34^ by GC/MS or HPLC and by incorporating Cl into the carbon surface (see XPS results).

On the other hand, GAC was characterized after cycles C1 and C3 to evaluate the influence of regeneration–resaturation cycles on the GAC properties. The BET surface area, pore volume, and the atomic surface concentration (by XPS) were measured for CAG after cycles C1 and C3 in R3 (Table 2) after regeneration (180 min), washing, and resaturation steps. Figure S7 collects the nitrogen adsorption–desorption isotherms obtained at −196 °C for these samples. The BET surface area and pore volume were calculated from these isotherms, and the results are summarized in Table 4.

**Table 4:** Characterization of GAC-R3 (Table 2) after First (C1) and Third (C3) Regeneration–Resaturation Cycles^a^

		GAC-S	C1			C3		
analytical technique	property	initial	R	R-W	R-W-S	R	R-W	R-W-S
N_2_ adsorption	*A*_BET_ (m^2^·g^–1^)	611	477	439	241	484	437	313
	*V*_P_ (cm^3^·g^–1^)	0.314	0.232	0.224	0.128	0.241	0.229	0.167
CO_2_ adsorption	*A*_DR_ (m^2^·g^–1^)	364	328	321	333	289	306	348
	*V*_DR_ (cm^3^·g^–1^)	0.146	0.132	0.128	0.133	0.116	0.123	0.139
XPS: atomic surface concentration	C (%)	89.29	81	82.77	87.13	77.25	78.94	79.01
	N (%)	0.24	0.78	1.15	0.22	–	0.63	0.69
	O (%)	8.33	16.48	14.6	15.00	17.56	17.11	17.94
	S (%)	0.16	0.72	0.25	0.16	0.65	0.24	–
	Cl (%)	1.08	1.02	1.24	0.9	4.54	2.31	1.2
TPD	CO (μmol·g^–1^)	413	1403	1413	1321	1597	1784	154
	CO_2_ (μmol·g^–1^)	76	446	341	337	669	714	556
	H_2_O (μmol·g^–1^)	367	506	797	609	606	628	590

a Properties were measured after 180 min of regeneration (R), after washing with Milli-Q water at 60 °C for 120 min (R-W), and after resaturation in 124-TCB (R-W-S).

The BET surface area and pore volume of GAC dropped from 871 m^2^·g^–1^ (see Table 3) to 611 m^2^·g^–1^ when the washed GAC (GAC-F) was saturated with 124-TCB. However, this value should be analyzed considering that GAC-S includes the adsorbed mass of 124-TCB (about 35% in GAC-F). After C1, the BET surface area of C1-R was 477 m^2^·g^–1^ and it was kept constant after the washing step (C1-R-W). Resaturation in 124-TCB (C1-R-W-S) causes a depletion of 50% in the BET surface area. The same trend was also found in C3. However, the differences in BET surface areas from C3-R-W and C3-R-W-S were lower than the one noticed in C1. The BET surface area after C3-R was almost the same as after C1-R, suggesting that the loss of surface area caused by incorporating the contaminant was recovered with the regeneration cycles. The corresponding nitrogen adsorption–desorption isotherms are shown in Figure S7.

The comparison between the BET surface area value in samples GAC-F-B8-C3b (see Table 3) and the corresponding value of R3-C3-R-W-S (shown in Table 4) suggested that the presence of 124-TCB on GAC protected the carbon surface against the PS attack. The same conclusion can be inferred from the data of PS consumption gathered in Figures 2 and 3b. The consumption of PS was also decreased when the 124-TCB was adsorbed on the GAC surface. Both circumstances suggest that PS preferably reacts with 124-TCB, keeping the apparent surface area practically constant after three regenerative/adsorption cycles.

This conclusion is also supported by the analysis of the atomic surface concentration and the presence of carbon–oxygen surface groups (given in Table 4) after C1 and C3 in run 3 (Table 2). First, a decrease in the carbon concentration upon oxidative attack by PS can be observed. However, this decrease is less drastic in GAC saturated in 124-TCB (Table 4) than that noticed for the unsaturated GAC (Table 3). Regarding the presence of oxygen surface groups, an increase in oxygen was observed due to the increase of acidic surface groups (carboxylic groups) on the carbon surface. The concentration of sulfur is maximum after regeneration, and this sulfur concentration is reduced after washing with water, indicating that this step is necessary to release the GAC surface.

The oxygen groups on the GAC surface increase with the regeneration (Table 4). However, the formation of carbon–oxygen surface groups, mainly as acid groups, is lower (approximately around 50%) than that noticed after the reaction of GAC-F with the oxidant (Table 3). As previously mentioned, the oxidative attack on the GAC surface in the presence of 124-TCB seems to be more selective to the pollutant, instead of leading to preferential oxidation of the carbon surface. On the other hand, some Cl incorporation into the carbon surface was also observed, even after the regeneration treatment. The analyses of the Cl 2p spectra show a unique band at 200.2 eV associated with the presence of organic Cl.

## 4 Conclusions

In the present work, the adsorption capacity recovery (RC) of a GAC, previously saturated with 124-TCB has been successfully carried out using PS activated with temperature. Three successive cycles of regeneration and saturation of GAC were accomplished, finding stable RC values and maintaining similar physicochemical properties of the adsorbent throughout the different reaction cycles. Between cycles, intermediate washes were implemented to remove the sulfur residues deposited during the regeneration operation. The application of these cycles ensured the promotion of the circular economy of GAC and the elimination of a highly toxic pollutant. In this sense, increasing the lifetime of the activated carbon in consecutive adsorption/oxidation cycles reduces the amount of carbon waste and the necessity of treating high amounts of activated carbons by conventional treatments.

In addition, the reaction between PS and GAC in the absence of 124-TCB adsorbed was investigated. This reaction generated mainly acidic groups on the carbon surface and reduced the apparent surface area of the carbon. The oxidation of the carbon competed with the abatement of 124-TCB. However, it was concluded that the oxidation of 124-TCB preferentially took place under the experimental conditions tested here. For saturated carbon, the sulfate radical attack was aimed at removing the 124-TCB present, avoiding the significant oxidation of the carbon surface of the adsorbent.

This work confirmed that the use of PS activated with temperature can be used to abate toxic chlorinated compounds adsorbed in GAC since the presence of the contaminant in the carbon surface favors the reaction rate and the reusability of the carbon. Further studies should be carried out to optimize the reaction conditions and to improve the efficiency of the oxidation reactions.

## Supporting Information Available

Scheme of the experimental setup in the saturation and saturation/regeneration steps; characterization of the fresh and washed carbon and their adsorption isotherms; PS conversion after successive cycles without and with washing of the GAC recovered between cycles using C_PS,0_: 166 mM; adsorption isotherms for GAC obtained after C1 and C3 of experiment B1 at 60 °C with 5 g·L^–1^ and 168 mM of initial PS; and adsorption isotherms for GAC-F, GAC-S, GAC-R3-C 1b (after regeneration, washing, and resaturation), and GAC-R3-C3b (after regeneration, washing, and resaturation) (PDF)
