# Supplementary material for: Regeneration of Granulated Spent Activated Carbon with 1,2,4-Trichlorobenzene Using Thermally Activated Persulfate
Source: Ind Eng Chem Res. 2022 Jun 28;61(27):9611–20. doi: 10.1021/acs.iecr.2c00440 (PMC9284557; doi:10.1021/acs.iecr.2c00440)
Supplement: Supplementary file 1 — ie2c00440_si_001.pdf [file ie2c00440_si_001.pdf]

**Title: REGENERATION OF GRANULATED SPENT ACTIVATED CARBON WITH 1,2,4-TRICHLOROBENZENE USING THERMALLY ACTIVATED PERSULFATE.**

**Authors:Andrés Sánchez-Yepes[a], Aurora Santos[a], Juana Mª Rosas[b], José Rodríguez-Mirasol[b], Tomás Cordero[b], David Lorenzo[a*].**

**(*) corresponding author: dlorenzo@quim.ucm.es**

[a] Universidad Complutense de Madrid, Departamento de Ingeniería Química y de Materiales, Madrid, 28040, Spain.

[b] Universidad de Málaga, Andalucía Tech, Departamento de Ingeniería Química, Málaga 29010, Spain.

**Keywords:** granular activated carbon, 1,2,4-trichlorobenzene, persulfate, regeneration.

**GAC SATURATION**

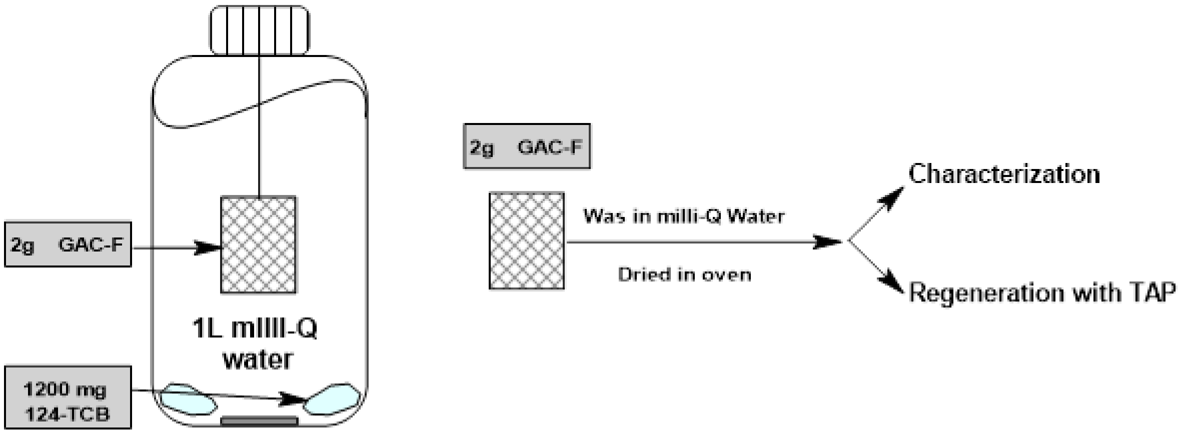


Figure S1 Diagram of the GAC saturation process at 124-TCB.

**REGENERATION / ADSORPTION CYCLES**

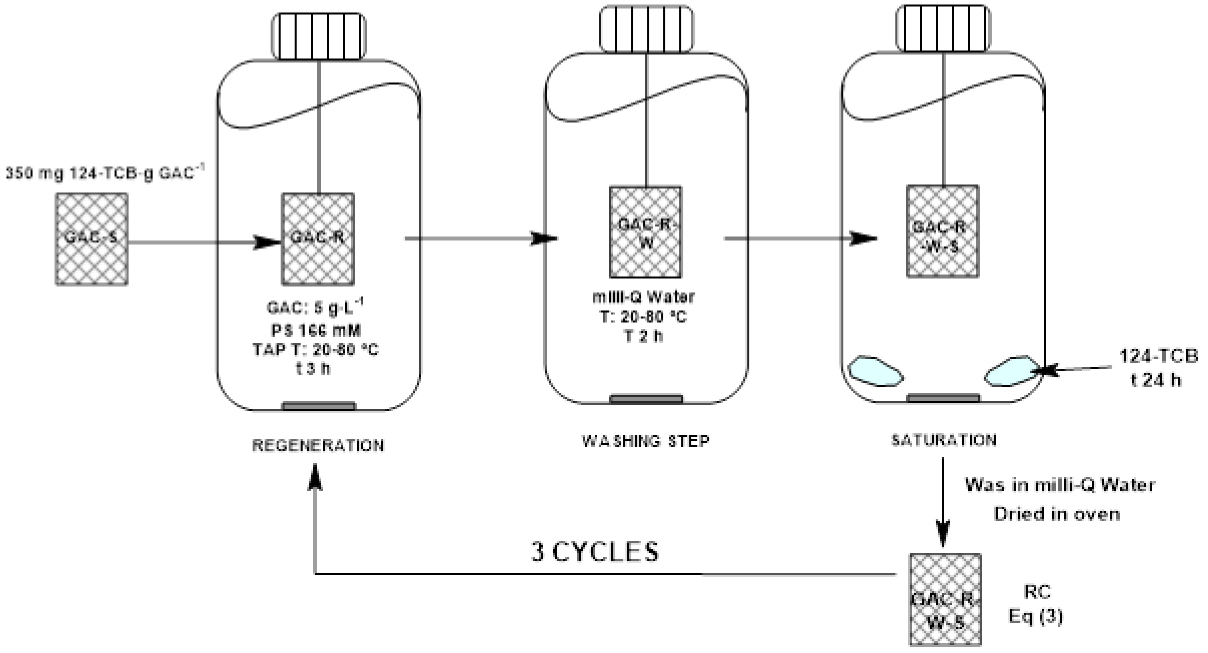


Figure S2 Diagram of the Regeneration and Adsorption operation.

Table S1 Characterisation results of fresh carbon, CAF, and fresh washed carbon, CAF-W.

| Analytical technique | Property | GAC-O | GAC-F |
|---|---|---|---|
| $N_2$ adsorption | $A_{BET}$ ($m^2 \cdot g^{-1}$) | 905 | 871 |
| | $V_P$ ($cm^3 \cdot g^{-1}$) | 0.42 | 0.39 |
| $CO_2$ adsorption | $A_{DR}$ ($m^2 \cdot g^{-1}$) | 435 | 459 |
| | $V_{DR}$ ($cm^3 \cdot g^{-1}$) | 0.174 | 0.184 |
| XPS: Atomic surface concentration | **C (%)** | 88.82 | 90.59 |
| | **N (%)** | 0.25 | 0.51 |
| | **O (%)** | 10.6 | 8.75 |
| | **S (%)** | 0.33 | 0.15 |
| TPD | $CO$ ($\mu mol \cdot g^{-1}$) | 290 | 349 |
| | $CO_2$ ($\mu mol \cdot g^{-1}$) | 18 | 41 |
| | $H_2O$ ($\mu mol \cdot g^{-1}$) | 50 | 73 |

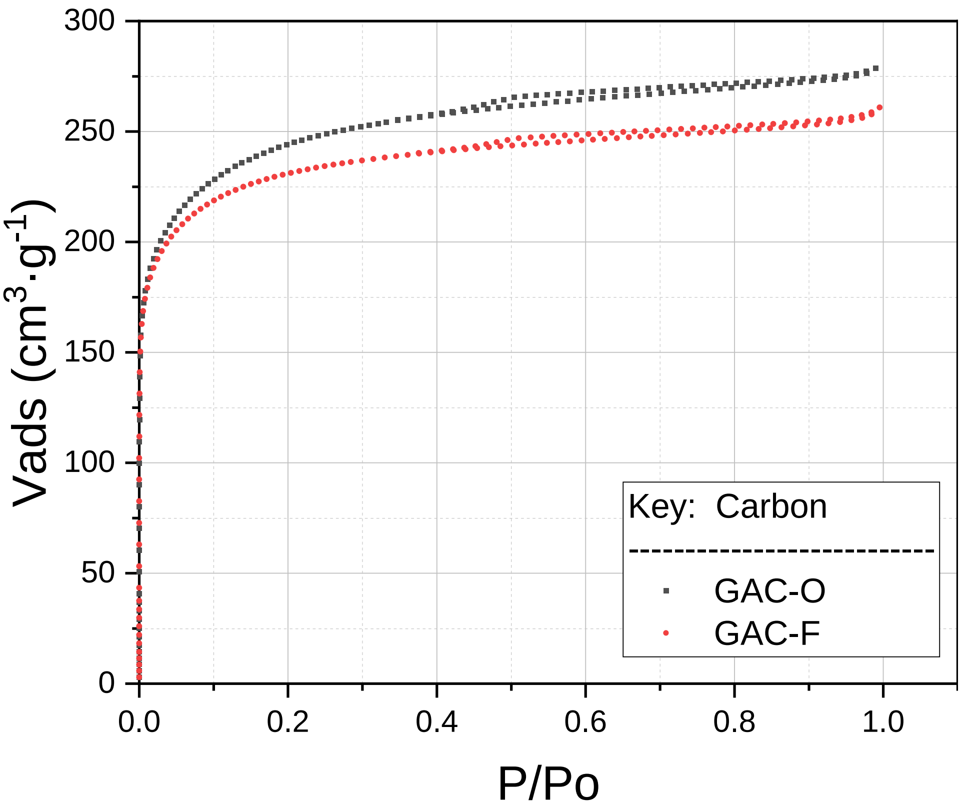


Figure S3 Adsorption Isotherms for GAC-O and GAC-F

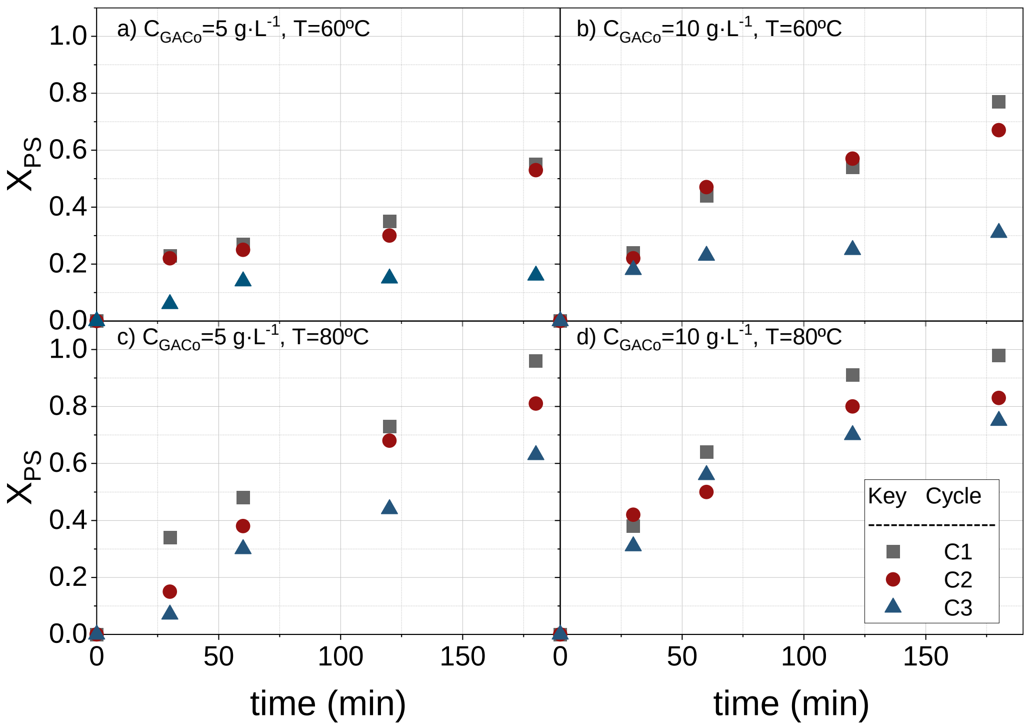


Figure S3. Persulfate conversion after successive cycles, without washing of the GAG recovered between cycles using $C_{PS,0}$: 166 mM.

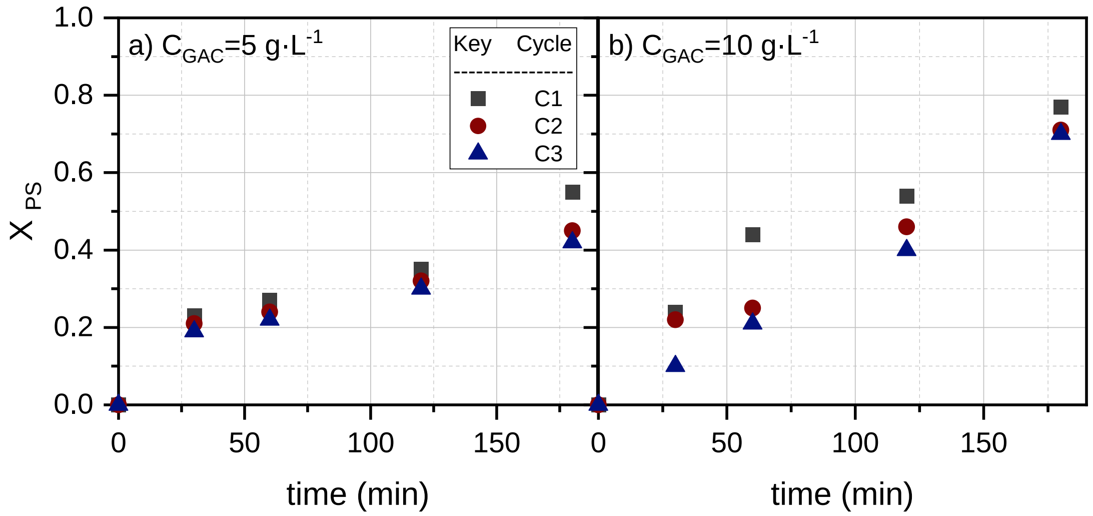


Figure S4. Persulfate conversion after successive cycles, washing of the GAG recovered between cycles (2 h at 60°C.) using $C_{PS,0}$: 166 mM and T=60ºC.

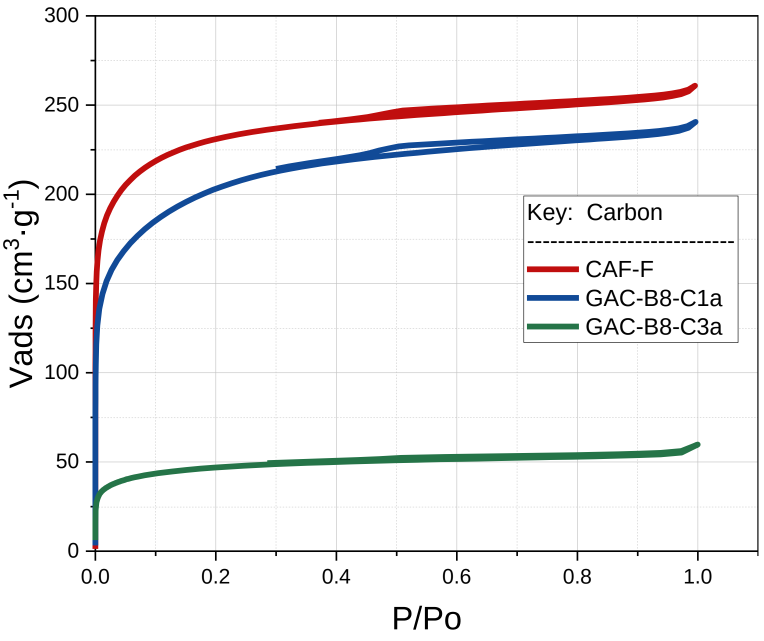


Figure S5. Adsorption Isotherms for GAC obtained after C1 and C3 of experiment B1 at 60 ºC with 5 $g \cdot L^{-1}$ and 168 mM of initial PS.

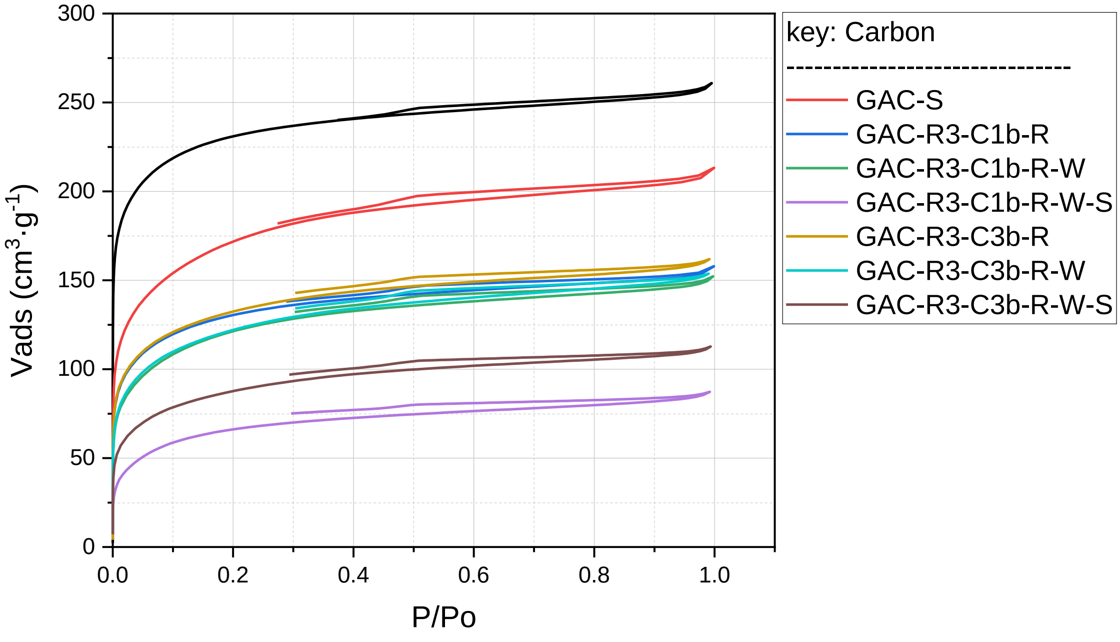


Figure S7 Adsorption Isotherms for GAC-F, GAC-S, GAC-R3-C1b (after regeneration, after washing, after resaturation) and GAC-R3-C3b (after regeneration washing, and resaturation)
